# ChIP-on-chip analysis of thyroid hormone-regulated genes and their physiological significance

**DOI:** 10.18632/oncotarget.7988

**Published:** 2016-03-08

**Authors:** I-Hsiao Chung, Hsuan Liu, Yang-Hsiang Lin, Hsiang-Cheng Chi, Ya-Hui Huang, Chang-Ching Yang, Chau-Ting Yeh, Bertrand Chin-Ming Tan, Kwang-Huei Lin

**Affiliations:** ^1^ Department of Biochemistry, College of Medicine, Chang Gung University, Taoyuan, Taiwan; ^2^ Molecular Medicine Research Center, Chang Gung University, Taoyuan, Taiwan; ^3^ Liver Research Center, Chang Gung Memorial Hospital, Linkou, Taoyuan, Taiwan; ^4^ Department of Biomedical Sciences, College of Medicine, Chang Gung University, Taoyuan, Taiwan; ^5^ Department of Neurosurgery, Chang Gung Memorial Hospital, Taoyuan, Taiwan; ^6^ Colorectal Section, Chang Gung Memorial Hospital, Linkou, Taoyuan, Taiwan

**Keywords:** thyroid hormone receptor, ChIP-on-chip, cell growth, ELF2

## Abstract

Triiodothyronine (T_3_) and its receptor (TR) modulate several physiological processes, including cell development, proliferation, differentiation and metabolism. The regulatory mechanism of T_3_/TR involves binding to the thyroid hormone response element (TRE) within the target gene promoter. However, the number of target genes directly regulated by TRα1 and the specific pathways of TR-regulated target genes remain largely unknown. Here, we expressed TRα1 in a HepG2 cell line and used chromatin immunoprecipitation coupled with microarray to determine the genes that are directly regulated by TRα1 and also involved in cell metabolism and proliferation. Our analysis identified E74-like factor 2 (ELF2), a transcription factor associated with tumor growth, as a direct target downregulated by T_3_/TR. Overexpression of ELF2 enhanced tumor cell proliferation, and conversely, its knockdown suppressed tumor growth. Additionally, ELF2 restored the proliferative ability of hepatoma cells inhibited by T_3_/TR. Our findings collectively support a potential role of T_3_/TR in tumor growth inhibition through regulation of ELF2.

## INTRODUCTION

Thyroid hormone receptors (TRs) are ligand-dependent transcription factors that mediate biological activities, such as cell growth, development and differentiation of the thyroid hormone (TH) [1–4]. Human TRs belong to a superfamily of nuclear receptors, and are encoded by *THRA* and *THRB* genes located on human chromosomes 17 and 3, respectively [5]. The two functional receptor isoforms, TRα1 and TRβ1, are expressed at different levels across various tissues and bind T_3_. TR binds as a monomer, homodimer, or heterodimer with retinoid X receptor (RXR) at thyroid hormone response elements (TREs) to regulate target gene transcription [6–8]. In T_3_-depleted conditions, TRs recruit nuclear corepressors for transcriptional repression of genes positively regulated by T_3_. Conversely, T_3_-bound TR undergoes conformational changes that result in release of co-repressors, allowing recruitment of nuclear receptor coactivators to facilitate transcriptional activation [9, 10]. A number of recent studies have attempted to characterize the functions and mechanisms underlying the positive or negative transcriptional regulation of TRβ1 [11–13]. However, limited information is available on genes directly regulated by TRα1 that are involved in critical pathways.

While several TRα1-regulated target genes have been identified in liver, their regulatory mechanisms and functional effects have not been reported to date [14]. A previous microarray study did not address whether these effects are directly or indirectly regulated by TRα1. Recently, the TRα1 and TRβ1 cistromes were analyzed in a neural cell line using overexpressed, tagged receptors, revealing that the two receptor isoforms share some overlap in binding sites but also have unique targets [15]. However, it remains to be established whether T_3_ can directly and specifically regulate TRα1–binding genes in hepatoma cell lines.

To characterize the TRα1 binding sites in a hepatoma cell line, we performed chromatin affinity precipitation coupled with microarray under T_3_ treatment conditions. Notably, T_3_ treatment enhanced TRα1 binding both positively and negatively at distinct genomic sites, and these changes were strongly correlated with those in the expression of associated genes. These results support a specific mechanism underlying the regulation of target genes by TRα1 whereby transcriptional changes are effected by T_3_ dictating differential binding of TRα1 through preferred motifs.

The E26 transformation-specific (ETS) family has been increasingly recognized as key regulators of cell differentiation, hormone responses and tumorigenesis in target tissues [16, 17]. The ETS family of genes is highly diverse, consisting of both transcriptional activators and repressors that mediate growth factor signaling and regulate gene expression through interactions with multiple protein partners [18]. ELF2, belonging to the ETS family, is associated with cell proliferation [19] and downregulated by T_3_/TR. In the current study, we focused on the role of ELF2 and mechanisms underlying its regulation by T_3_/TR in a hepatoma cell line. Based on the collective findings, we propose that T_3_/TR suppresses cell proliferation through downregulation of ELF2 in HCC.

## RESULTS

### ChIP-on-chip analysis of gene binding in HepG2-TRα1 cells

**Figure 1:**
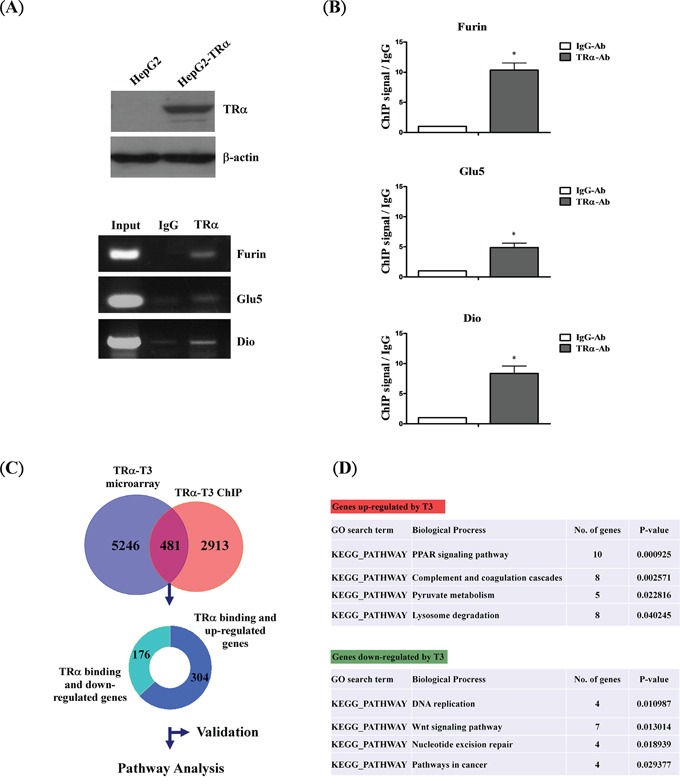
Schematic diagram of ChIP-on-chip analysis **A.** Western blot analysis of TR expression in extracts of TRα1-overexpressing cell lines. The positions of 47 kDa TRα1 are indicated. ChIP assay demonstrating that TRα1 is recruited to the TRE regions of positive control genes (*FURIN*, *GLU5* and *DIO*) using RT-PCR and **B.** q-RT-PCR, respectively. **C.** Overlapping genes between ChIP-on-chip and oligonucleotide microarray in T_3_-treated conditions. **D.** Functional pathways of genes directly regulated by T_3_/TR. Differences were analyzed using one-way ANOVA, *P < 0.05.

A HepG2 cell line stably expressing high levels of wild-type TRα1 (HepG2-TRα1) was established for analyses (Figure 1A, upper panel). Well-known TR-binding genes, such as *Furin* [21], *Glu5* [22] and *Dio* [23], were employed to determine direct regulation by T_3_/TR using the ChIP assay. TR proteins were clearly associated with the TRE region within *Furin*, *Glu5* and *Dio* promoters *in vivo* (Figure 1A, lower panel and Figure 1B). TRα1 was recruited to the TRE-binding site whereas control IgG produced only background levels. Under similar conditions, the ChIP-on-chip assay was used to assess the global and direct binding genes of T_3_/TR. Overall, more than two thousand (2913) genes showed direct binding, 481 of which were enriched from ChIP-on-chip coupled with oligonucleotide microarray of genes in hepatoma cultures treated with T_3_ (5246 genes) (Figure 1C). Among these, 304 up- and 176 down-regulated genes were directly bound and modulated by T_3_/TR.

To determine the functions of the 481 identified genes, bioinformatics pathway analysis (DAVID) was performed. Upregulated genes (304) were involved in cell metabolism pathways, such as PPAR-α signaling, pyruvate metabolism and lysosome degradation, while downregulated genes (176) were linked to cancer-associated pathways, such as Wnt signaling, DNA replication and repair. We additionally validated direct binding and regulation by T_3_/TR of the two known up- and downregulated genes, *PPAR-α* [24] and *c-MYC* [25–27], respectively (Figure S1). Our results support the theory that TRα1 protein binds the promoter regions of these target genes for transcriptional regulation.

### Genes directly bound and positively regulated by T_3_/TR

**Figure 2:**
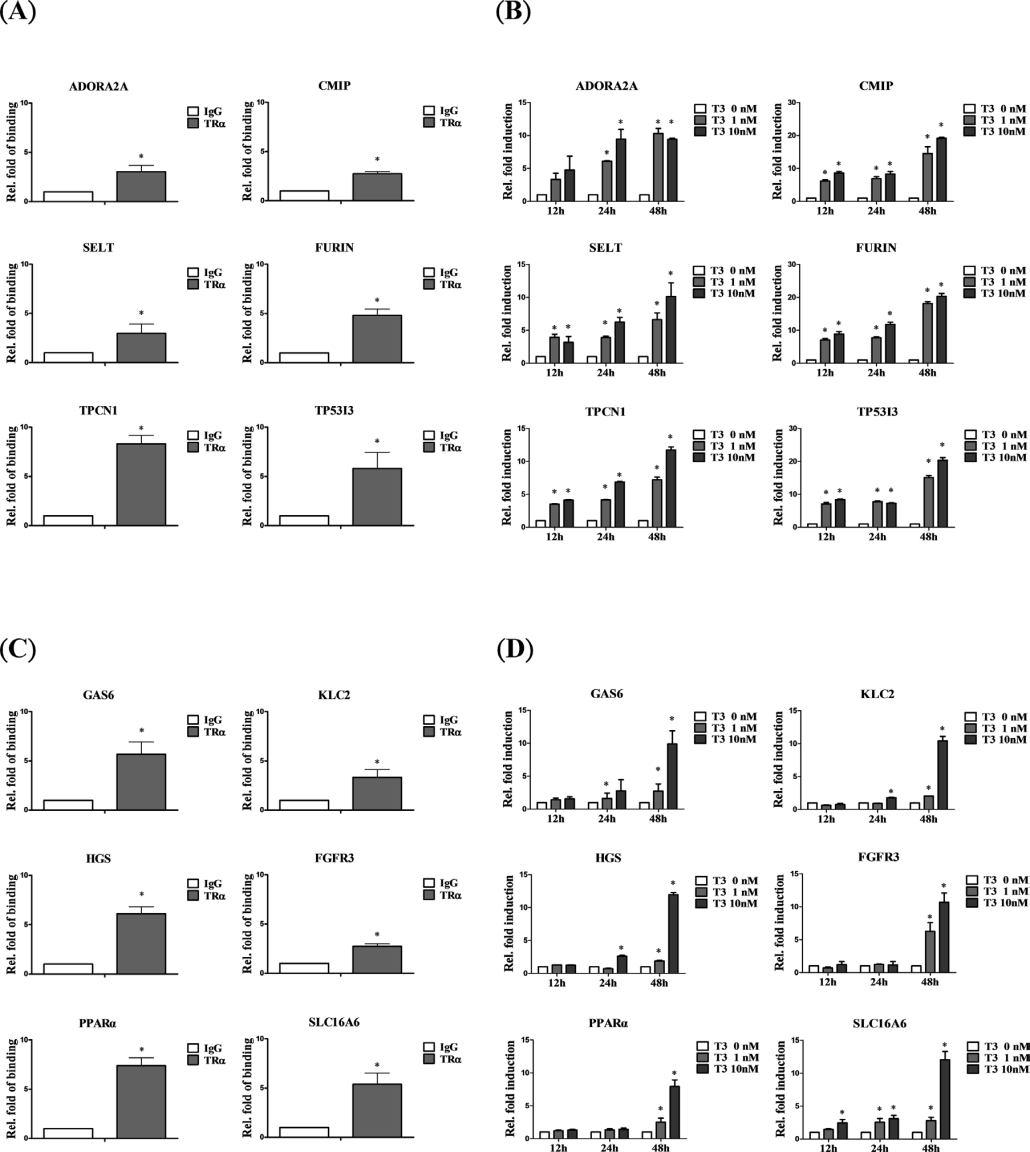
Genes positively regulated by T_3_/TR **A.** Relative binding fold, **B.** q-RT-PCR assessment of expression of T_3_/TR-upregulated genes with rapid responses (12-48 h) in the absence or presence of 1 or 10 nM T_3_ in HepG2-TRα1cells **C, D.** T_3_/TR-upregulated genes with delayed responses. Differences were analyzed using one-way ANOVA, *P < 0.05.

To validate ChIP-on-chip data, the top 50 ranking genes were selected. Overall, 92% of genes (46/50) that directly interact with TR were verified. However, among these genes, 84.78% (39/46) were truly regulated by T_3_/TR. Furthermore, 28 of 33 (84.85%) genes were upregulated. Symbolic genes, such as *ADOR2A2*, *CMIP*, *SELT*, *FURIN*, *TPCN1* and *TP53I3* directly interacted with and were rapidly and positively regulated by T_3_/TR (Figure 2A, 2B). Other genes, such as *GAS6*, *KLC2*, *HGS*, *FGFR3*, *PPAR-α* and *SLC16A6* bound directly and were positively regulated, but at a slower rate (Figure 2C, 2D).

### Genes directly bound and negatively regulated by T_3_/TR

**Figure 3:**
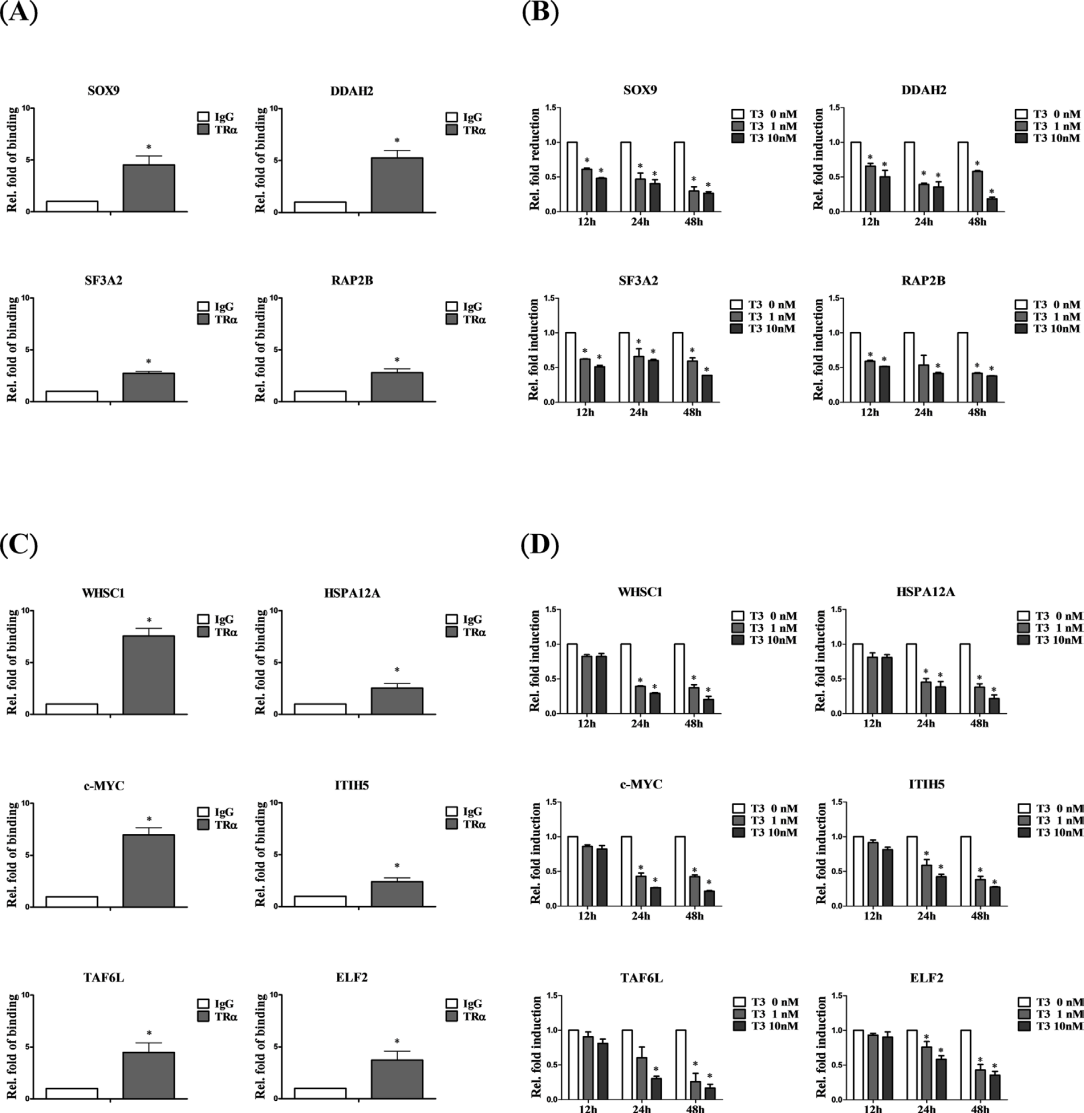
Genes negatively regulated by T_3_/TR **A.** Relative binding fold, **B.** q-RT-PCR assessment of expression levels of T_3_/TR- downregulated genes with rapid responses (12-48 h) in the absence or presence of 1 or 10 nM T_3_ in HepG2-TRα1cells **C, D.** T_3_/TR-downregulated genes with delayed responses. Differences were analyzed using one-way ANOVA, *P < 0.05.

According to the ChIP-on-chip database, 11 of 13 (84.85%) genes bound directly and were rapidly downregulated, including *SOX9*, *DDAH2*, *SF3A2* and *RAP2B* (Figure 3A, 3B), while *WHSC1*, *HSPA12A*, *c-MYC*, *ITIH5*, *TAF6L* and *ELF2* bound directly but were negatively regulated at a slower rate (Figure 3C, 3D). Taken together, the results support the high accuracy of our database and confirm the identities of specific genes directly regulated by T_3_/TR in hepatoma cells.

### T_3_/TR supresses cell proliferation *in vitro* and *in vivo*

**Figure 4:**
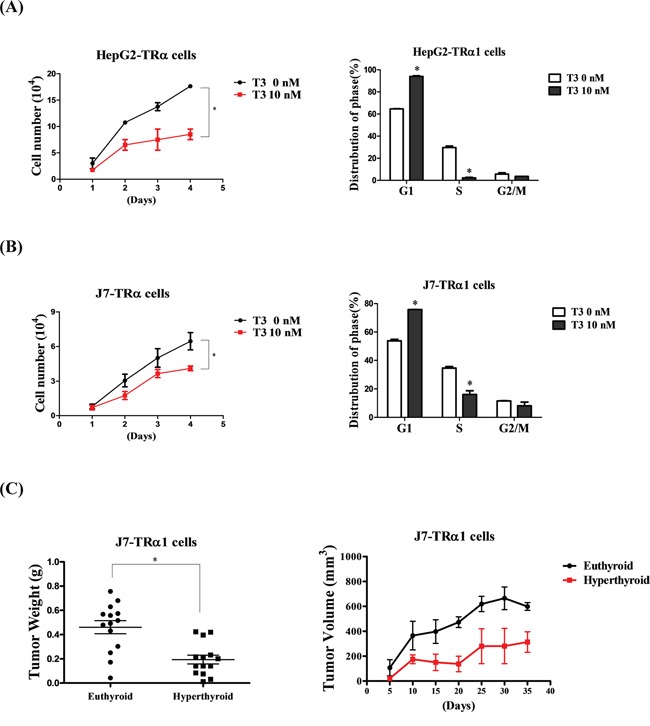
T_3_/TR suppresses cell proliferation and the cell cycle *in vitro* and *in vivo* Cell growth ability and cell cycle were analyzed in two TRα1-overexpressing **A.** HepG2 and **B.** J7 cell lines in the absence or presence of 10 nM T_3_. The number of cells was counted to determine proliferation activity. The cell cycle stage was detected via flow cytometry. **C.** Tumor weights and volumes of nude mice injected with J7-TRα1 cells treated with high levels of T_3_ (hyperthyroid) and administered normal drinking water (euthyroid). Differences were analyzed using one-way ANOVA, *P < 0.05.

Pathway analysis suggested that T_3_/TR suppresses the proliferation of cancer cells. To further examine this theory, HepG2 and J7 cell lines stably expressing high levels of wild-type TRα1 (HepG2-TRα1 and J7-TRα1, respectively) were established. Notably, the proliferation ability of HepG2-TRα1 and J7-TRα1 cells was significantly suppressed upon T_3_ stimulation (Figure 4A, 4B left panel). Moreover, the cell cycle of HepG2-TRα1 and J7-TRα1 cells was arrested at the G1 phase following T_3_ treatment (Figure 4A, 4B right panel). To verify whether the *in vitro* effect of T_3_/TR can be replicated *in vivo*, nude mice were injected with J7-TRα1 cells following by T_3_ stimulation. Higher T_3_ levels (hyperthyroid conditions) induced suppression of tumor weight and volume, compared to that in control mice (euthyroid conditions) (Figure 4C). Both our *in vitro* and *in vivo* findings support the potential utility of T_3_/TR as a target for effective suppression of cancer cell proliferation.

### T_3_/TR suppresses cell growth via downregulation of ELF2

**Figure 5:**
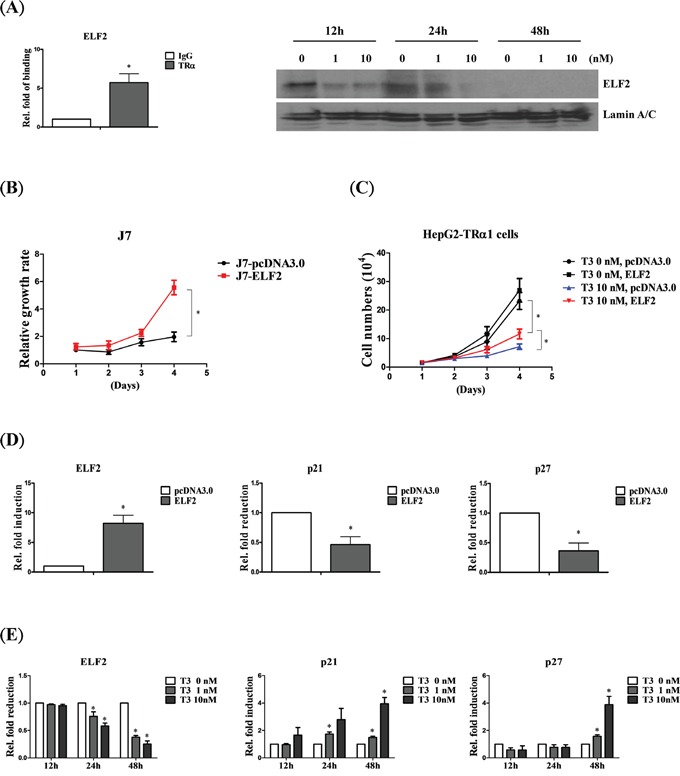
ELF2 is negatively regulated by T_3_/TR to suppress cell proliferation **A.** ChIP assay demonstrating that TRα1 is recruited to the *ELF2* TRE (left panel). Western blot analysis of ELF2 protein expression in stable HepG2-TRα1 cell lines at 12-48 h in the absence or presence of 1 and 10 nM T_3_ (right panel). **B.** Cell proliferation analysis via cell counting in ELF2-overexpressing and control J7 cell lines. **C.** ELF2 overexpression rescues the proliferation activity of HepG2-TRα1 cells at 48 h in the absence or presence of 10 nM T_3_. q-RT-PCR detection of p21 and p27 in ELF2-overexpressing, compared to vector control-expressing **D.** J7 cells or **E.** HepG2-TRα1 cells in similar T_3_ conditions. Differences were analyzed using one-way ANOVA, *P < 0.05.

ELF2 is a transcription factor that promotes cancer cell proliferation [28]. According to our database, ELF2 is directly bound and downregulated by T_3_/TR (Figure 5A). To determine the mechanism underlying the association of T_3_/TR with ELF2 function in proliferation, ELF2 was overexpressed in J7 and HepG2-TRα1 cell lines. J7 cells overexpressing ELF2 displayed significantly increased proliferation, compared with control cells (Figure 5B), along with markedly decreased expression levels of two cell growth inhibitory genes, p21 [29, 30] and p27 [31, 32] (∼0.4 to 0.6 and ∼0.3 to 0.5-fold, respectively) (Figure 5D). Moreover, both p21 and p27 were downregulated by T_3_/TR (Figure 5E). Notably, the proliferative ability of HepG2-TRα1 control cells (T_3_ 0 nM, pcDNA3.0) was markedly suppressed under T_3_ stimulation (T_3_ 10 nM, pcDNA3.0), but restored upon ELF2 re-expression (HepG2-TRα1-ELF2) (Figure 5C). The observed T_3_-induced inhibition of cancer cell growth and ELF2 expression supports the hypothesis that T_3_/TR suppresses tumor proliferation via ELF2 regulation.

### ELF2 depletion suppresses cell growth through p21 and p27 signaling

**Figure 6:**
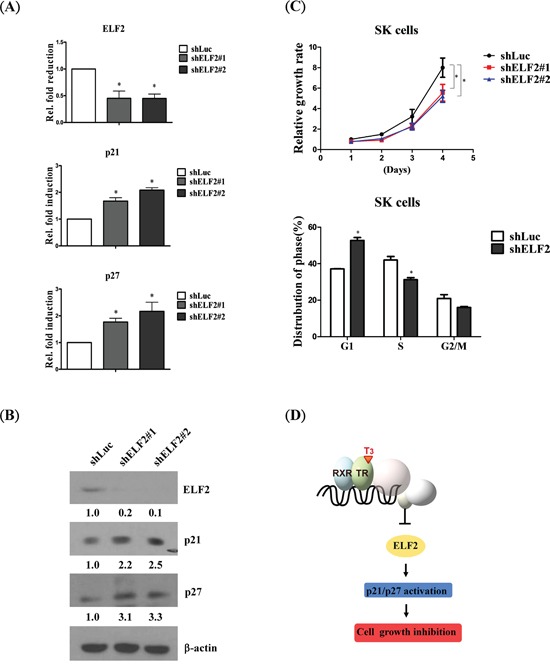
ELF2 depletion suppresses cell proliferation via p21 and p27 activation Detection of ELF2, p21 and p27 expression in ELF2-depleted (shELF2#1, shELF2#2) and control (shLuc) SK-Hep1 cells using **A.** q-RT-PCR and **B.** western blot, respectively. **C.** Cell proliferation analysis in ELF2-depleted (shELF2#1, shELF2#2) and control (shLuc) SK-Hep1 cells (upper panel). Cell cycle stages of ELF2-depleted (shELF) and control (shLuc) SK-Hep1 cells detected via flow cytometry (lower panel). **D.** Schematic diagram showing that T_3_/TR negatively regulates ELF2 to suppress cell proliferation via p21/p27 activation. Differences were analyzed using one-way ANOVA, *P < 0.05.

To determine the consequences of ELF2 depletion, SK-HEP1 control cell lines and shLuc and ELF2 knockdown lines (shELF2#1 and shELF2#2) were established (Figure 6A, upper panel). After depletion of ELF2, proliferation of SK-HEP1 cells was decreased, compared with control cells (Figure 6C, upper panel), confirming the ability of ELF2 to accelerate tumor cell growth. Moreover, ELF2-depleted cells were arrested at the G1 phase, consistent with the effects of T_3_ (Figure 6C, lower panel). Accordingly, we further examined whether p21 and p27 activation are implicated in ELF2-depleted phenotypes. Marked upregulation of p21 and p27 was observed in ELF2-depleted cells (shELF2#1 and shELF2#2), compared with control cells (shLuc) (Figure 6A, 6B). Our findings suggest that stimulation of p21 and p27 in hepatoma cells is mediated via ELF2 repression. Based on the collective data, we conclude that T_3_/TR-mediated downregulation of ELF2 suppresses cell proliferation via activation of p21 and p27 (Figure 6D).

## DISCUSSION

Our ChIP-on-chip experiments demonstrated that TRα1 binds to multiple regions across the hepatoma genome, with a high percentage of binding sites located within genes. Examination of differential TRα1 binding in hyperthyroid conditions revealed that many sites are bound with increased affinity, depending on the presence of ligand. Importantly, integration with microarray data showed that TRα1 binding significantly correlated with expression patterns of target genes transcriptionally regulated by T_3_. In this study, we identified TRα1 binding sites associated with 50 genes in HepG2-TRα1 cells. Binding sites may be located in different genomic contexts (both upstream and downstream of the gene and within introns) and may not adhere to conventional sequence motifs (Supplementary Table). Our findings support the notion that the thyroid hormone can regulate several genes through direct binding by TR.

The Ets family of proteins consists of a number of transcription factors that share a conserved winged helix-turn-helix DNA binding domain (Ets domain). Ets factors are critical mediators of a variety of cellular processes, including development, differentiation, growth, and transformation [18]. Some Ets genes may be involved in specific chromosomal translocations in different cancer types [33], suggesting an oncogenic role. Indeed, overexpression of several Ets genes has been reported in cancers of the thyroid, pancreas, liver, colon, lung, and leukemia [34]. ELF2 belongs to the Elf subfamily of Ets transcription factors together with ELF1 and myeloid elf-1-like factor (MEF), and regulates a set of genes in B cells and myeloid cells [35]. Moreover, ELF2 physically interacts with acute myeloid leukemia 1 (AML1), a frequent target for chromosomal translocations in leukemia [36]. ELF2 expression is increased in endothelial cells in response to hypoxia and angiopoietin-1 [37]. While these findings suggest an oncogenic role of ELF2, its precise role in tumorigenesis has not been clarified as yet. In the current study, we showed that ELF2 is modulated by T_3_ at both mRNA and protein levels. Our experiments confirmed that T_3_ regulates *ELF2* at the transcriptional level and TR proteins directly bind TRE of the *ELF2* promoter region. Notably, cell lines overexpressing ELF2 displayed higher proliferation. Moreover, T_3_-mediated suppression of ELF2 occurred via p21 and p27 activation, leading to inhibition of cancer cell progression.

The liver is a typical target organ of thyroid hormones (THs). Equal amounts of TRα1 and TRβ1 proteins are expressed in human hepatocytes. Recent studies have suggested that long-term hypothyroidism is associated with HCC, independent of other major HCC risk factors [38]. Hypothyroidism is characterized by insufficient production of THs and inappropriate TR action and is a possible risk factor in human cancers. However, no results directly showing that thyroid status is associated with tumor progression have been reported as yet. Earlier studies demonstrated that liver function abnormalities return to normal once primary thyroid pathology is recognized and treated [39]. Subsequently, T_3_ was shown to mediate apoptosis and accelerate necrosis in liver cells [40]. These results suggest that hypothyroidism increases the risk of liver cancer through decreased apoptosis in the liver lesion process. Another group showed that hypothyroidism is associated with high risk of HCC in women [38]. However, the mechanisms linking hypothyroidism with HCC need to be clarified with further studies on different population groups.

In conclusion, T_3_ induces an increase in TRα1 binding, leading to either positive or negative regulation of target genes. The results of this study provide new insights into the mechanisms of transcriptional regulation by TRα1: T_3_-mediated negative regulation of ELF2 may contribute to reduction of hepatoma cell growth through subsequent activation of the cell cycle checkpoint.

## MATERIALS AND METHODS

### Cell culture

Human hepatoma cells, HepG2, SK-Hep1 and J7, were routinely cultured at 37°C in a humidified atmosphere of 95% air and 5% CO_2_ in Dulbecco's modified Eagle's medium (DMEM) supplemented with 10% fetal bovine serum (FBS). HepG2 and J7 cell lines were stably transfected with TRα1 (HepG2-TRα1 and J7-TRα1).

### Chromatin immunoprecipitation (ChIP) assay

HepG2-TRα1 cells treated with 10 nM T_3_ for 24 h or left untreated were harvested and cross-linked with 1% formaldehyde for 10 min at room temperature in DMEM. Reactions were terminated with the addition of 0.125 M glycine. Subsequently, cell lysates were washed three times with PBS and resuspended in lysis buffer (150 mM NaCl, 5 mM EDTA, 50 mM Tris (pH 8.0), 0.1% SDS and 0.1% sodium deoxycholate) containing three protease inhibitors (1 mM PMSF, aprotinin, and leupeptin). Cell lysates were sonicated with a Misonix Sonicator 3000 Homogenizer (Mandel Scientific Company Inc., Guelph, ON, Canada) to disrupt chromatin. Sonicated DNA was between 200 and 1000 bp in length. Products were precleared with 60 μl protein A/G agarose (Sigma Chemicals, St. Louis, MO) for 2 h at 4°C. Complexes were immunoprecipitated with anti-TR (kindly provided by the laboratory of Dr. S-Y Cheng at the National Cancer Institute) and anti-IgG antibodies (R&D Systems, Inc., Minneapolis, MN). Enriched targets were hybridized to promoter microarrays (Welgene Biotech, ChIP-on-chip microarray) spanning −8 kb to +2 kb of the transcription start site (TSS) of 35000 genes. The promoter fragments of target gene containing the TRE region were detected via q-RT-PCR. All of primers were listed at Supplementary Table.

### Cloning of ELF2

cDNA was synthesized from total RNA (1 μg) using Superscript II reverse transcriptase (Invitrogen, Carlsbad, CA) and oligo (dT) primers. *ELF2* was amplified from cDNA by polymerase chain reaction (PCR) using the primer pair 5′-ATG GCG ACG TCT CTG CAT GAG GGA C-3′ (forward) and 5′-TTA TTT CTC ACA TGT CAC TAG TCC T-3′ (reverse), and the following thermocycling conditions: 30 cycles at 95°C for 1 min, 58°C for 1 min, and 72°C for 2 min. The *ELF2* open reading frame was ligated into the pcDNA 3.0 expression vector, and the resulting construct was sequenced to confirm the presence of the gene.

### Establishing J7 cell lines stably overexpressing ELF2

The J7 cell line, grown in 10-cm cell culture dishes, was transfected with the ELF2 expression plasmid using the Lipofectamine reagent (Invitrogen). After 24 h, transformants were selected from transfected cells by growing in medium containing the antibiotic G418 (400 μg/ml). Expression of ELF2 protein in the selected clones was detected using Western blot analysis.

### shRNA-mediated ELF2 knockdown

Short hairpin RNA (shRNA) sequences targeting ELF2 were purchased from the National RNAi Core Facility (Institute of Molecular Biology, Academia Sinica, Taiwan). The SK-HEP1 line was transiently transfected with shRNA targeting the endogenous *ELF2* gene using the Turbofect reagent (Invitrogen). ELF2 repression was confirmed by Western blot analysis.

### Immunoblot analysis

Total cell lysates were prepared, and protein concentrations determined with the Bradford assay kit (Pierce Biotechnology, Rockford, IL). Equivalent amounts of proteins were fractionated on a 10% sodium dodecyl sulfate (SDS)-polyacrylamide gel. Separated proteins were transferred to nitrocellulose membrane (pH 7.9, Amersham Biosciences Inc., Piscataway, NJ), blocked with 5% non-fat powdered milk, and incubated with specific anti-ELF2 (GeneTex; GTX104851), anti-p21 (abcam; ab109520), anti-p27 (abcam; ab32034), anti-c-MYC (GeneTex; GTX103436) and anti-PPARα (GeneTex; GTX101098) primary antibodies at −20°C overnight. After washing, membranes were incubated with HRP-conjugated anti-mouse, anti-rabbit or anti-goat IgG secondary antibody, as appropriate, for 1 h at room temperature. Immune complexes were visualized using an enhanced chemiluminescence (ECL) detection kit (Amersham) and Fuji X-ray film.

### *In vitro* proliferation assays

The influence of ELF2 on the cell proliferation abilities of J7-ELF2 and SK-HEP1-ELF2-depleted cells were determined. The proliferation abilities under T_3_ (10nM) condition in HepG2-TRα1 cells and restored by ELF2 expression were identified *in vitro* as described previously [20]. Briefly, cell density was adjusted to 10^6^ cells/ml, and 100 μl of the suspension seeded on 24 well plate. The medium was DMEM with 10% fetal bovine serum (FBS). After incubation for 1-4 days at 37°C, cells harvested were examined via MTT assay or cell counting. Experiments were performed at least three times.

### *In vivo* proliferation assays

Similar conditions were employed with nude mice containing various T_3_ levels induced via injection of J7-TRα1 cells [7]. Mice were divided into two groups, specifically, Group A (euthyroid) comprising control mice given normal drinking water and Group B (hyperthyroid) administered drinking water augmented with T_3_ (2 mg/L) (Sigma Chem. Co., St. Louis, MO) after inoculation of tumor cells. Mice were sacrificed about 1 month after injection, their livers and lungs removed for tumor biopsy, and the T_3_ and TSH levels determined. The T_3_ and TSH levels in sera of euthyroid mice (Group A) were 45.5 ng/dl and 0.246 mIU/ml, while those in sera of hyperthyroid mice (Group B) were 619 ng/dl and 0.008 mIU/ml, respectively. Tumor volume was calculated using the following equation: length × height × width. All procedures were performed under sterile conditions in a laminar flow hood. Animal experiments were performed in accordance with the United States National Institutes of Health guidelines and Chang-Gung Institutional Animal Care and Use Committee Guide for the Care and Use of Laboratory Animals.

### Cell cycle assay

For detection of cell cycle phases, HepG2-TRα1 cells were starved in serum-free medium for 24 h, followed by incubation in medium with or without T_3_. After 24 h, cells were harvested with trypsin and washed twice with cold phosphate-buffered saline. SK-HEP1 control and SK-HEP1-ELF2-depleted cells were harvested without T_3_ condition. Subsequently, cells were fixed with 70% ethanol for 1 h at −20°C and incubated in 0.5% Triton X-100/phosphate-buffered saline containing 0.05% DNase-free RNase for 1 h at 37°C. Nuclei were stained with propidium iodide (50 μg/ml) after treatment with 0.5% Triton X-100/PBS containing 0.05% DNase-free RNase, and the DNA content was analyzed by flow cytometry using the fluorescence-activated cell sorting Aria cell sorting system (Becton Dickinson). The percentage of cells in each phase of the cell cycle was determined using the Modfit LT program (Verity Software House).

### Statistical analysis

Data are expressed as mean values ± SEM of at least three experiments. Statistical analysis was performed using Student's *t* test and one-way ANOVA. *P* values < 0.05 were considered statistically significant.

## SUPPLEMENTARY FIGURES AND TABLES




