# Supplementary material for: ChIP-on-chip analysis of thyroid hormone-regulated genes and their physiological significance
Source: Oncotarget. 2016 Mar 8;7(16):22448–59. doi: 10.18632/oncotarget.7988 (PMC5008372; doi:10.18632/oncotarget.7988)
Supplement: Supplementary file 1 [file oncotarget-07-22448-s001.pdf]

SUPPLEMENTARY FIGURE AND TABLE

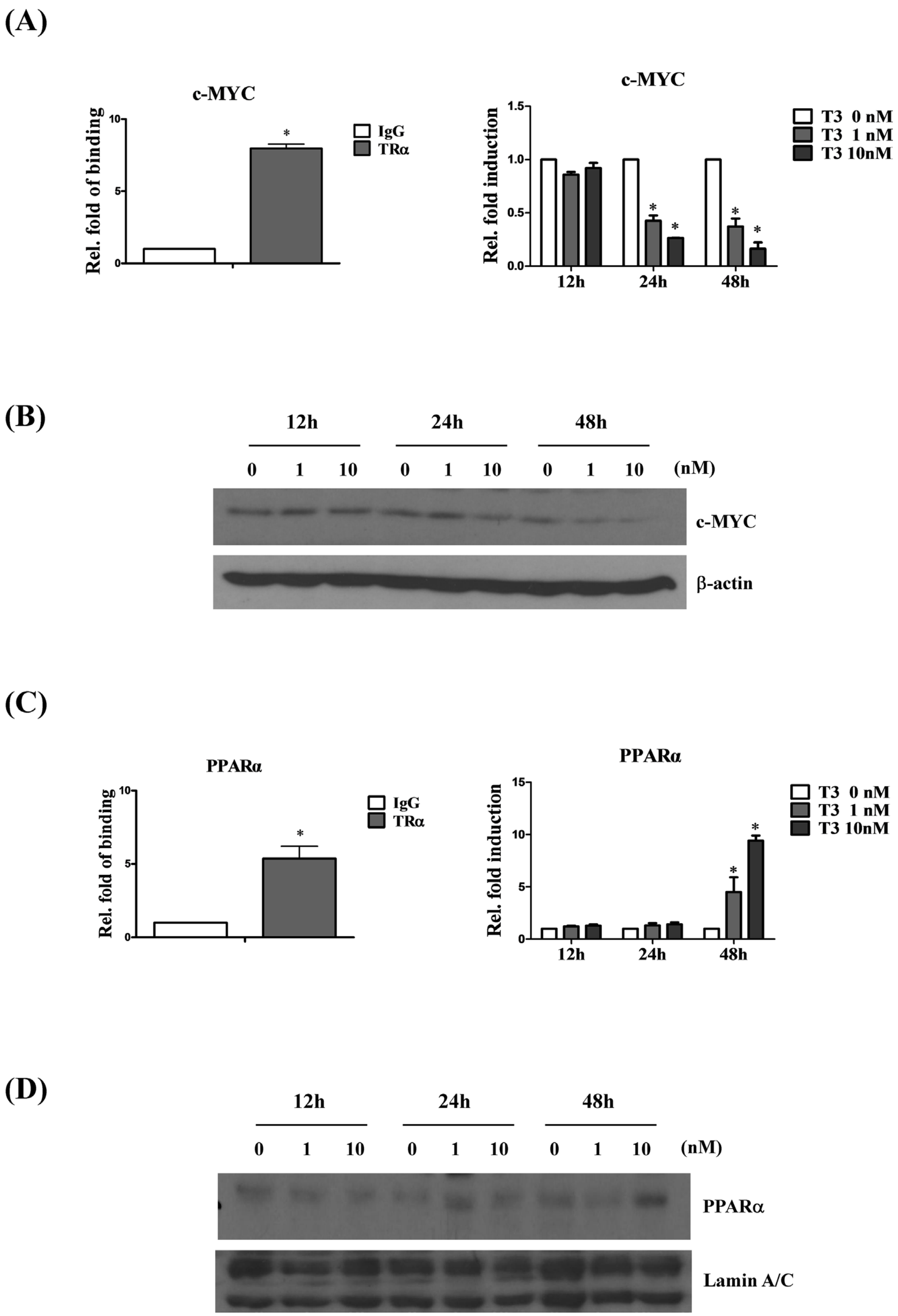

Supplementary Figure S1: T_3/TR regulates c-MYC and PPAR-α expression in HepG2-TRα1 cells. Expression and binding of T_3 regulates c-MYC and PPAR-α in cell extracts of overexpressed TRα1 cell lines, as determined via **A, C.** q-RT-PCR and **B, D.** western blotting. The positions of 55 kDa c-MYC and 56 kDa PPAR-α are indicated after treatment with 0, 1, and 10 nM T_3 for 12-48 h.

Supplementary Table 1: The primers of q-RT-PCR (For ChIP samples)

See Supplementary File 1
